# Association between the atherogenic index of plasma and testosterone deficiency in American adults: a cross-sectional study from NHANES 2011–2016

**DOI:** 10.3389/fendo.2025.1531221

**Published:** 2025-05-08

**Authors:** Yanghao Tai, Bin Chen, Yingming Kong, Jiwen Shang

**Affiliations:** ^1^ Third Hospital of Shanxi Medical University, Shanxi Bethune Hospital, Shanxi Academy of Medical Sciences Tongji Shanxi Hospital, Taiyuan, China; ^2^ Department of Urology, Shanxi Bethune Hospital, Shanxi Academy of Medical Science, Tongji Shanxi Hospital, Third Hospital of Shanxi Medical University, Taiyuan, China

**Keywords:** AIP, testosterone deficiency, lipid metabolism, population-based studies, NHANES

## Abstract

**Background:**

A common pathophysiological association between lipid metabolism and sex hormone levels has been revealed in recent research. The atherogenic index of plasma (AIP) is the marker currently used to evaluate metabolism. The purpose of this research was to discover the relationship between the AIP and testosterone deficiency (TD) in a nationwide representative population.

**Methods:**

Data from the National Health and Nutrition Examination Survey (NHANES) database from 2011 to 2016 were utilized in this cross-sectional research. The formula, lg [TG (mmol/L)/HDL-C(mmol/L)], was applied to determine the AIP. Total serum testosterone levels were used to define TD. Our researcher utilized smoothed curve fitting and multivariate logistic or linear regression analysis to inspect the relationship between AIP and TD among adult males. The consistency of these results was examined in various population subgroups.

**Results:**

In total, 1,198 individuals (28.6%) were stratified into the TD group. We observed statistically significant differences (P values < 0.05) in the TD population for all variables. After correcting for potential confounders, our researchers discovered a strong positive relationship between the AIP and the probability of developing TD. With each additional unit of the AIP, the incidence of TD increased by 2.81-fold in adult males. Subgroup analyses showed the correlations for the majority of the subgroups remained stable. However, marital status, CKD, smoking, and alcohol consumption may modify this association.

**Conclusions:**

A higher AIP is correlated with a lower level of testosterone in adult males. This correlation may be altered by factors including marriage, chronic kidney disease, alcohol, and smoking consumption.

## 1 Background

The main source of testosterone, an important class of sex hormones, is the testes in the male reproductive system, and in particular, the interstitial cells within the testes are responsible for synthesizing and releasing this hormone in large quantities. In addition to this, the body’s adrenal glands and ovaries are also involved in the production of testosterone (1). As a result, testosterone levels in men are generally significantly higher than in women and are essential for maintaining male secondary sexual characteristics and fertility (2). Research has revealed that the highest levels of testosterone production in men are during puberty and early adulthood, followed by a gradual decrease with age. After the age of 40, there is an annual decline of 0.4%–2.6%, and by the age of 80, testosterone concentrations may be 20% to 50% lower in comparison to younger ages (3–5). The role of testosterone is not limited to sex characterization and individual growth and development but is also involved the maintenance of a variety of physiological functions (6). In the United States, approximately 20% to 50% of men suffer from testosterone deficiency (TD) (7), a prevalent health problem that not only affects reproductive health and sexual function, but also leads to psychological and physiological disorders, cognitive dysfunction, muscular atrophy, osteoporosis, and related metabolic disorders such as depression (8–11). As a key regulator of men’s health, testosterone modulates a number of biological processes that are critical to men’s health. Reduced or insufficient serum testosterone levels have been associated with an increased risk of several cardiovascular diseases, such as hypertension, stroke, and coronary heart disease (12–14). In addition, TD has a pivotal role in the development of metabolic diseases, which include dyslipidemia, diabetes, and obesity (15, 16). Research suggests that deficient testosterone levels may be correlated with the incidence and seriousness of these metabolic diseases. Amid the increasing worldwide demographic shift towards an older population, the associated problem of lower testosterone levels has gradually attracted widespread public attention. Epidemiological statistics reveal that the average prevalence of TD in males above the age of 50 is approximately 7%, with the incidence increasing significantly with age (17, 18). Therefore, identifying and establishing biomarkers that can accurately predict changes in testosterone levels is of paramount importance for clinical practice, not only to help prevent a range of physical and mental disorders caused by testosterone deficiency but also to have a significant impact on improving the overall life satisfaction of patients.

Current medical research has found that individuals with abnormal lipid metabolism and obesity are often accompanied by lower testosterone levels, which rebound significantly when these metabolic problems are effectively treated. These findings reveal a strong link between testosterone levels and an individual’s metabolic health status (19–22). The plasma atherosclerotic index (AIP) is a new lipid biomarker that signifies the characteristics and severity of abnormal lipid metabolic procession (23). The AIP is strongly associated with atherosclerotic load and cardiovascular events (24–28). The AIP reflects the ratio of triglyceride (TG) to high-density lipoprotein cholesterol (HDL-C) and represents the dimensions of lipoprotein molecules, which indicates the disease-causing potential and distinctive nature of dyslipidemia more effectively than TG or HDL-C levels by themselves (29, 30). Since testosterone levels are associated with metabolic status and obesity, and the AIP reflects the lipid metabolic status of an individual, abnormalities in the AIP may suggest changes in testosterone levels. However, to our knowledge, the predictive value of the AIP in diagnosing testosterone deficiency among adult individuals remains unclear. Our research aimed to explore the correlation between the AIP and the prevalence of testosterone deficiency among US adults based on a nationally representative sample.

## 2 Materials and methods

### 2.1 Data sources

The 2011–2016 National Health and Nutrition Examination Survey (NHANES) datasets were the original information resource for the present analysis. The NHANES database provides comprehensive data on each participant’s laboratory tests, disease information, human examinations, and population characteristics. The reliability of the data in this database stems from the fact that it is collected based on a nationwide representative sample in the United States via a comprehensive investigation, using the most advanced and reliable techniques of information collection and organizational and statistical methods for data analysis. This data is subsequently subjected to additional analysis and utilized to study risk factors for a variety of diseases. A sophisticated multi-stage probabilistic approach was used in this study to ensure the representativeness and accuracy of the samples used. The qualification of human subjects in NHANES was accessible via the National Center for Health Statistics (NCHS) Ethics Review Board and each participant provided signed informed consent.

### 2.2 Research population

**Figure 1:**
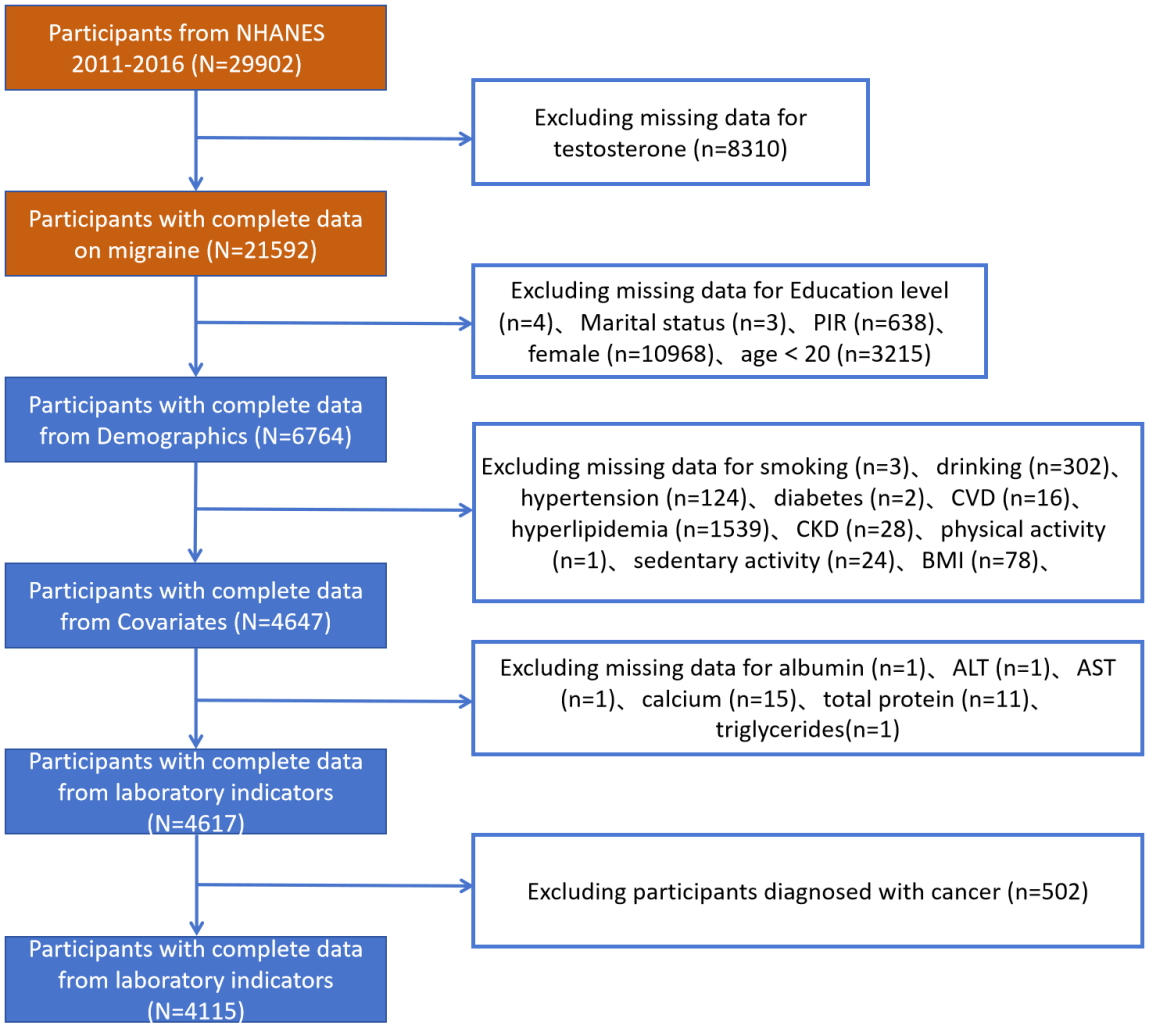
Flowchart depicting participant selection in the study.

The researchers selected 29,902 participants from NHANES 2011–2016.The researchers requested that the dataset be a usable dataset containing information on TD, and the relevant variables needed to calculate the AIP. The researchers applied the exclusion criterion to the following groups: participants aged <20 years; those lacking sociodemographic data, confounders, and incomplete information on TD; and participants with incomplete data required for the AIP calculation. A total of 4,115 participants with comprehensive information participated in this cross-sectional investigation after the exclusion of participants who met the above criteria (**Figure 1**). This study applied the WTMEC2YR assessment weights to correct for sampling bias and improve the generalizability of the results.

### 2.3 Definition of the exposure variable

The AIP was regarded in the current investigation as an exposure variable, calculated by lg [TG (mg/dL)/HDL-C(mg/dL)]. TG values and HDL-C were assessed using a Cobas 6000 biochemistry instrument. We treated the AIP as both a categorical variable and a continuous variable for correlation studies for the purpose of further investigating the relationship between TD and AIP.

### 2.4 Definition of the outcome variable

The researchers considered the prevalence of TD and the level of testosterone as the outcome variables. Total testosterone in serum was measured using an isotope dilution liquid chromatography-tandem mass spectrometry (ID-LC-MS/MS) method developed by the Centers for Disease Control and Prevention (CDC) for routine analysis. The total testosterone level for the diagnosis of TD according to the Association of American Urology was <300 ng/dL.

### 2.5 Covariates

Factors that may have an impact on the association between TD and the AIP were regarded as covariates in this investigation. The covariates considered in this study included the sociodemographic factors of age (≤40, 41–60, or >60 years), educational level (high school graduate/GED or equivalent, college graduate or above, or less than 12th grade), ethnicity (other Hispanic, other races, Mexican American, non-Hispanic Black, or non-Hispanic white), marital condition (living alone, married, or living with partner), and family poverty income ratio (PIR) (≤1.0, 1.1-4.0, or >4.0). The researchers also included the health-related factors of diabetes, sedentary behavior, physical activity, hypertension, hyperlipidemia, chronic kidney disease (CKD), and cardiovascular diseases (CVDs) (all described as yes/no). According to questionnaire information, self-reported disease was also considered, including diabetes. A diagnosis of cardiovascular disease, hypertension, and hyperlipidemia was regarded as the basis of a self-reported history. The definition of cardiovascular disease includes angina, congestive heart failure, stroke, heart attack, and coronary heart disease. A diagnosis of CKD was defined as urinary Albumin-to-Creatinine Ratio (uACR)>30 mg/g or eGFR <60 ml/min/1.73 m². Specialized medical technicians took careful measurements of an individual’s body mass index (BMI) in a Mobile Examination Center. BMI is classified as overweight (25–30 kg/m²), obesity (≥30 kg/m²), or normal (<25 kg/m²). Physical activity is defined as a questionnaire response to moderate recreational activities. According to the responses to the questionnaire, sedentary behavior was defined as more than 360 minutes of sitting or lying down in a day at work, home, or school, while sleep time was excluded. PIR is the measurement of socioeconomic position, described as the rate of income to the United States Census Bureau’s poverty level for a family. Individuals are classified as present smokers, never smokers, or former smokers based on the “Do you personally use cigarette or tobacco?” and “Smoked more than 100 cigarettes in your lifetime” questions. Based on the “Drink at least twelve times in a lifetime or a year” question, the definition of a alcohol drinker includes current, never, and former alcohol drinkers. Laboratory data included albumin, liver enzymes, urea nitrogen, serum creatinine, calcium, phosphorus, sodium, potassium, total bilirubin, uric acid, and creatinine.

### 2.6 Statistical methods

Continuous and categorical variables were used to characterize participants at baseline. Categorical variables are described by numbers and percentages. Continuous variables are described in terms of mean ± standard deviation. Weighted chi-square tests and t-tests were conducted for comparisons of baseline characteristics among these participants. By assessing the corrected odds ratio (OR), β, and 95% confidence interval (CI), the study conducted a weighted logistic or linear regression model to explore the connections between TD, testosterone levels, and AIP. Three models were used by us with varying degrees of modification for covariates (Model 1, unadjusted for covariates; Model 2, modified for marital status, education, age, ethnicity, and PIR; and Model 3, adjustments for marital status, smoking status, BMI, PIR, education, alcohol consumption, physical activity, age, ethnicity, diabetes, hypertension, CVD, CKD, sedentary behavior, hyperlipidemia, and laboratory test indicators). In all three models, continuous variable AIP was used by our researchers. In addition, the dose-response relationship between AIP and the prevalence of the TD and testosterone level was evaluated by utilizing the restricted cubic spline (RCS) curves. This multiple logistic or linear regression model includes the continuous and categorical models. The AIP was divided into quartiles, and then the linear trends were calculated by considering the median value of every subgroup as a continuous variable. In addition, our researchers conducted subgroup analyses of general information, sedentary behavior, smoking status, disease condition, physical activity, alcohol consumption, and their interactions to examine whether there were different associations between subgroups. All statistical analyses in this investigation were conducted utilizing R 4.3.3 and SPSS 26.0. A bilateral P-value < 0.05 was recognized as statistically significant.

## 3 Result

### 3.1 Baseline characteristics of participants

The study recruited 29,902 individuals from NHANES 2011–2016. **Figure 1** displays the flowchart for the exclusion and inclusion of individuals. Participants with incomplete information for testosterone were excluded and the remaining 21,592 participants were retained. Participants with incomplete information for age <20 years, sociodemographic data, and covariates were excluded, and the remaining 6,764 participants were retained. In addition, after excluding participants who had incomplete data required for AIP calculations and disease condition, the remaining 4,115 participants were considered in our investigation. The characteristics of the participants involved in this investigation at baseline are presented in **Table 1**. In total, 1,198 individuals (28.6%) were stratified into the TD group. We observed statistically significant differences (P-value < 0.05) in the TD population for all variables.

**Table 1:** Baseline characteristics of participants based on testosterone deficiency.

Characteristic	N	Participant no. (weighted, %)		P-value
		With TD	Without TD	
N	4,115	1,198 (28.6%)	2,917 (71.4%)	
Age				<0.001
20–40	1,243	300 (27.1%)	943 (34.9%)	
41–60	1,586	477 (48.4%)	1,109 (42.9%)	
61–80	1,286	421 (24.5%)	865 (22.2%)	
Race				<0.001
Mexican American	540	164 (8.0%)	376 (7.2%)	
Other Hispanic	397	119 (5.3%)	278 (5.1%)	
Non-Hispanic white	1,648	500 (70.7%)	1,148 (69.7%)	
Non-Hispanic Black	867	232 (8.0%)	635 (9.6%)	
Other Race - Including Multi-Racial	663	183 (8.0%)	480 (8.4%)	
Education level				<0.001
Less than 12th grade	845	266 (14.2%)	579 (12.2%)	
High school graduate/GED or equivalent	904	261 (21.4%)	643 (21.1%)	
College graduate or above	2,366	671 (64.4%)	1,695 (66.6%)	
Marital status				<0.001
Married and living with partner	2,781	878 (74.9%)	1,903 (67.8%)	
Living alone	1,334	320 (25.1%)	1,014 (32.2%)	
PIR				<0.001
≤1.0	764	222 (11.4%)	542 (11.8%)	
1.1–4.0	2,133	622 (46.0%)	1,511 (46.8%)	
> 4.0	1,218	354 (42.5%)	864 (41.4%)	
BMI				<0.001
<25	1,020	149 (11.3%)	871 (27.1%)	
25–30	1,587	399 (32.9%)	1,188 (41.9%)	
≥30	1,508	650 (55.9%)	858 (31.1%)	
Smoking				< 0.001
Never	1,947	565 (49.4%)	1,382 (50.6%)	
Past	1,268	437 (35.9%)	831 (27.7%)	
Now	900	196 (14.7%)	704 (21.7%)	
Drinking				<0.001
Never	347	111 (8.0%)	236 (6.6%)	
Past	318	100 (6.2%)	218 (5.4%)	
Now	3,450	987 (85.8%)	2,463 (88.0%)	
Diabetes				<0.001
Yes	677	296 (17.0%)	381 (9.7%)	
No	3,322	856 (80.1%)	2,466 (88.0%)	
Pre-diabetes	116	46 (2.9%)	70 (2.3%)	
Hypertension				<0.001
No	2,169	521 (49.7%)	1,648 (60.6%)	
Yes	1,946	677 (50.3%)	1,269 (39.4%)	
Hyperlipidemia				<0.001
No	1,755	407 (38.6%)	1,348 (48.5%)	
Yes	2,360	791 (61.4%)	1,569 (51.5%)	
CVD				<0.001
Yes	520	192 (12.3%)	328 (9.1%)	
No	3,595	1006 (87.7%)	2,589 (90.9%)	
CKD				< 0.001
Yes	785	290 (16.4%)	495 (12.1%)	
No	3,330	908 (83.6%)	2422 (87.9%)	
Moderate recreational activities				<0.001
Yes	1,850	505 (47.2%)	1,345 (50.4%)	
No	2,265	693 (52.8%)	1,572 (49.6%)	
Sedentary activity				<0.001
Yes	2,364	767 (68.1%)	1,597 (57.9%)	
No	1,751	431 (31.9%)	1,320 (42.1%)	
eGFR (ml/min/1.73 m²)	4,115	88.88 ± 25.29 (92.42 ± 23.43)	91.56 ± 23.55 (93.71 ± 21.66)	<0.001
Albumin (g/L)	4,115	43.05 ± 3.27 (43.53 ± 3.16)	43.89 ± 3.20 (44.36 ± 3.08)	<0.001
ALT (U/L)	4,115	31.23 ± 20.79 (33.11 ± 20.82)	29.17 ± 32.64 (29.19 ± 29.15)	<0.001
AST (U/L)	4,115	27.64 ± 13.52 (28.31 ± 14.71)	28.23 ± 25.20 (27.74 ± 21.04)	<0.001
ALP (U/L)	4,115	69.24 ± 23.67 (67.52 ± 26.55)	66.58 ± 20.98 (64.60 ± 19.85)	<0.001
BUN (mmol/L)	4,115	5.43 ± 2.42 (5.32 ± 1.97)	5.15 ± 1.98 (5.14 ± 1.71)	<0.001
Calcium (mmol/L)	4,115	2.35 ± 0.09 (2.35 ± 0.09)	2.36 ± 0.09 (2.36 ± 0.08)	<0.001
Creatinine (umol/L)	4,115	94.07 ± 55.92 (89.98 ± 42.01)	90.46 ± 41.58 (87.90 ± 29.41)	<0.001
Gamma glutamyl transferase (U/L)	4,115	35.66 ± 57.84 (34.64 ± 48.29)	32.86 ± 42.17 (31.32 ± 37.60)	<0.001
Phosphorus (mmol/L)	4,115	1.19 ± 0.19 (1.19 ± 0.19)	1.18 ± 0.18 (1.18 ± 0.18)	<0.001
Total bilirubin (umol/L)	4,115	11.12 ± 4.58 (11.38 ± 4.51)	12.39 ± 5.56 (12.71 ± 5.89)	<0.001
Total protein (g/L)	4,115	71.40 ± 4.67 (70.77 ± 4.6)	71.85 ± 4.69 (71.28 ± 4.45)	<0.001
Uric acid (umol/L)	4,115	375.63 ± 87.00 (376.07 ± 82.92)	354.73 ± 75.14 (355.33 ± 69.48)	<0.001
HDL-C (mmol/L)	4,115	1.13 ± 0.33 (1.12 ± 0.31)	1.28 ± 0.37 (1.29 ± 0.37)	<0.001
Triglycerides (mmol/L)	4,115	2.50 ± 1.79 (2.57 ± 1.74)	1.78 ± 1.48 (1.76 ± 1.39)	<0.001
AIP	4,115	0.27 ± 0.36 (0.29 ± 0.35)	0.06 ± 0.35 (0.06 ± 0.35)	<0.001

Continuous variables were presented as mean with standard deviation (mean t S), and categorical variables were expressed as proportion. Continuous variables were analyzed via one-way ANOVA; categorical variables were analyzed using the Chi-square test or the Fisher’s exact test, and P-value less than 0.05 was considered statistically significant.

### 3.2 Association between AIP and TD

The crude model revealed the prevalence of TD increased 3.94-fold (OR 4.94, 95%CI 4.05~6.02, P < 0.001) for every unit addition in the AIP. Model 2 was adjusted for sociodemographic indicators and showed a 4.43-fold (OR 5.43, 95%CI 4.41~6.69, P < 0.001) increase in prevalence of TD in adult males. Model 3 was further controlled for disease status, with every unit addition of the AIP significantly increasing the prevalence of TD by 2.81-fold (OR 3.81, 95% CI 3.00~4.82, P < 0.001).

As shown in **Table 2**, each additional unit of the AIP was also categorized by quartile and compared to the first quartile as the reference. In the crude model, compared with the lowest Q1, the highest Q4 had a 4.36-fold increase in the prevalence of TD (OR 4.36, 95% CI 3.54~5.36). In Model 2, after an adjustment for sociodemographic factors, the risk of TD was increased 4.69-fold (OR 4.69, 95% CI 3.77~5.82) compared with Q1. In Model 3, the incidence was increased 3.13-fold, after the total adjustment (OR 3.13, 95% CI 2.46~3.98).

**Table 2:** Weighted regression models and trend tests elucidating the association between AIP and testosterone deficiency.

AIP	Testosterone deficiency OR (95% CI)					
	Model 1	P-value	Model 2	P-value	Model 3	P-value
Continuous	4.94 (4.05~6.02)	<0.001	5.43 (4.41~6.69)	<0.001	3.81 (3.00~4.82)	<0.001
Quantile						
Q1	1.00 (Reference)		1.00 (Reference)		1.00 (Reference)	
Q2	1.46 (1.17~1.83)	<0.001	1.50 (1.20~1.88)	<0.001	1.22 (0.96~1.55)	0.099
Q3	2.73 (2.21~3.37)	<0.001	2.86 (2.30~3.56)	<0.001	2.08 (1.65~2.63)	<0.001
Q4	4.36 (3.54~5.36)	<0.001	4.69 (3.77~5.82)	<0.001	3.13 (2.46~3.98)	<0.001
P for trend		<0.001		<0.001		<0.001

Model 1 was not adjusted.

Model 2 was adjusted for age, race, education level, marital status, and PIR.

Model 3 was adjusted for age, race, education level, marital status, PIR, BMI, disease condition, physical activity, sedentary behavior, smoking status, alcohol use, disease, and laboratory test indicators.

### 3.3 Association between AIP and total testosterone

This crude model showed that total testosterone decreases by 154.07 (β =-154.07, 95% CI -168.56~-139.58, P<0.001) for every unit increase in the AIP. Model 2 was adjusted for sociodemographic factors and showed a 155.89 decrease in total testosterone (β =-155.89, 95%CI -170.83~-140.96, P<0.001). Model 3 further controlled for disease status, with every unit addition of the AIP significantly increasing the total testosterone by 111.97 (β = -111.97, 95% CI -127.89~-96.06, P<0.001).

**Figure 2:**
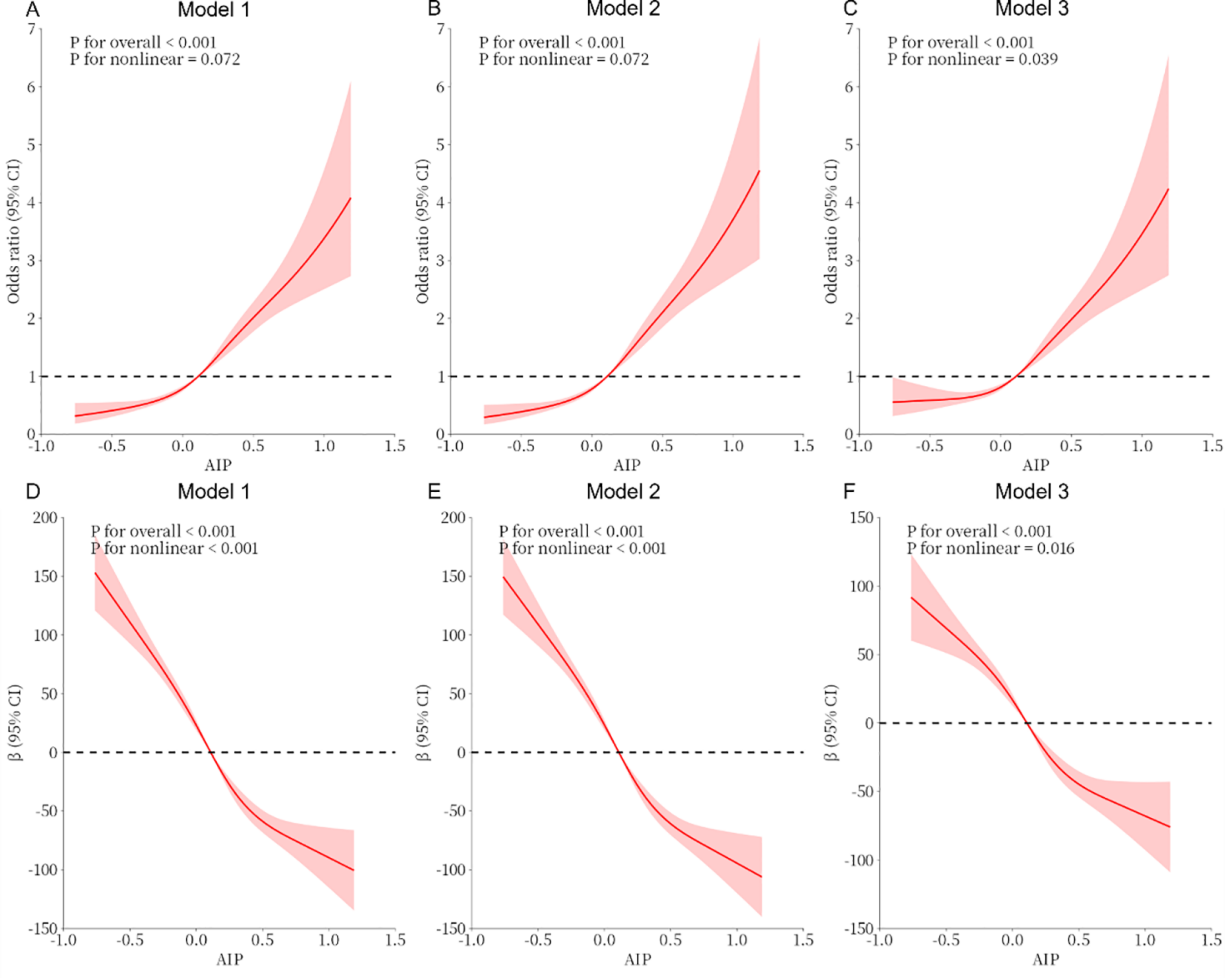
**(A–C)** Analysis of the measured response relationship between AIP and testosterone deficiency; **(D–F)** Analysis of the measured response relationship between AIP and testosterone levels. Model 1 was not adjusted. Model 2 was adjusted for age, race, education level, marital status, and PIR. Model 3 was adjusted for age, race, education level, marital status, PIR, BMI, disease condition, physical activity, sedentary behavior, smoking status, alcohol use, disease, and laboratory test indicators. The solid red line indicates the odds ratio or β and the red shaded area indicates the 95% confidence interval (CI).

As shown in **Table 3**, the total testosterone in the Q4 group decreased by 104.78 compared with the Q1 group under the same conditions (β = -104.78, 95% CI -120.89~-88.66, P <0.001). In all models, a significant dose-response trend was found with the increase in the AIP (P for trend < 0.001). In **Figure 2**, the constructed RCS curves show the dose-response relationship between AIP and TD or testosterone levels.

**Table 3:** Weighted regression models and trend tests elucidating the association between AIP and testosterone levels.

AIP	Testosterone levels β (95% CI)					
	Model 1	P-value	Model 2	P-value	Model 3	P-value
Continuous	-154.07 (-168.56~-139.58)	<0.001	-155.89 (-170.83~-140.96)	<0.001	-111.97 (-127.89~-96.06)	<0.001
Quantile						
Q1	0.00 (Reference)		0.00 (Reference)		0.00 (Reference)	
Q2	-54.90 (-70.00~-39.81)	<0.001	-53.31 (-68.39~-38.23)	<0.001	-34.27 (-48.86~-19.68)	<0.001
Q3	-110.96 (-126.05~-95.87)	<0.001	-110.49 (-125.73~-95.25)	<0.001	-77.34 (-92.53~-62.16)	<0.001
Q4	-146.87 (-161.96~-131.79)	<0.001	-147.35 (-162.79~-131.91)	<0.001	-104.78 (-120.89~-88.66)	<0.001
P for trend		<0.001		<0.001		<0.001

Model 1 was not adjusted.

Model 2 was adjusted for age, race, education level, marital status, and PIR.

Model 3 was adjusted for age, race, education level, marital status, PIR, BMI, disease condition, physical activity, sedentary behavior, smoking status, alcohol use, disease, and laboratory test indicators.

### 3.4 Subgroup analysis

Analyzed by weighted logistic regression, the relationship between the incidence of TD and AIP for each layered indicator is reported in **Table 4**. For each unit addition in the AIP, the incidence of TD increased 3.73-fold (OR 4.74, 95% CI 3.57 ~ 6.29) in participants married and living with a partner. However, for participants living alone, the incidence of TD increased by 76% (OR 1.76, 95% CI 1.14 ~ 2.71). In addition, we observed significant changes in CKD. The tiered analysis of disease status showed a 2.91-fold increase for participants without CKD (OR 3.91, 95% CI 2.99 ~ 5.11) and a 1.33-fold increased risk for individuals with hypertension (OR 2.33, 95% CI 1.44 ~ 3.79). Regarding smoking and alcohol consumption, the risk was 1.95-fold increased for participants who currently smoked (OR 1.95, 95% CI 1.17 ~ 3.24) and 3.29-fold increased for participants who currently consumed alcohol (OR 3.29, 95% CI 2.56 ~ 4.22). The results of the stratified analysis remained reliably stable between the remaining groups except for marital status, CKD, smoking, and alcohol consumption.

**Table 4:** Subgroup analysis of the association between the AIP and testosterone deficiency.

Subgroup	[OR (95% CI)]	P-value	P for interaction	Subgroup	[OR (95% CI)]	P-value	P for interaction
Age			0.133	CKD			**<0.001**
20–40	4.83 (2.97 ~ 7.84)	<0.001		No	3.91 (2.99 ~ 5.11)	<0.001	
41–60	3.21 (2.25 ~ 4.59)	<0.001		Yes	2.33 (1.44 ~ 3.79)	<0.001	
61–80	3.44 (2.24 ~ 5.28)	<0.001		Moderate recreational activities			0.423
Race			0.223	Yes	3.88 (2.69 ~ 5.60)	<0.001	
Mexican American	6.26 (3.13 ~ 12.54)	<0.001		No	3.26 (2.41 ~ 4.42)	<0.001	
Other Hispanic	2.90 (1.22 ~ 6.87)	0.016		Hypertension			0.090
Non-Hispanic white	4.06 (2.79 ~ 5.91)	<0.001		No	4.55 (3.20 ~ 6.47)	<0.001	
Non-Hispanic Black	2.56 (1.51 ~ 4.35)	<0.001		Yes	2.83 (2.06 ~ 3.87)	<0.001	
Other Race - Including Multi-Racial	4.80 (2.48 ~ 9.29)	<0.001		Hyperlipidemia			0.466
Education level			0.228	No	3.48 (2.33 ~ 5.19)	<0.001	
Less than 12th grade	3.27 (1.97 ~ 5.45)	<0.001		Yes	3.64 (2.73 ~ 4.86)	<0.001	
High school graduate/GED or equivalent	3.22 (1.97 ~ 5.26)	<0.001		Smoking			**0.005**
College graduate or above	3.82 (2.77 ~ 5.27)	<0.001		Never	4.84 (3.39 ~ 6.91)	<0.001	
Marital status			**0.001**	Past	3.48 (2.29 ~ 5.29)	<0.001	
Married and living with partner	4.74 (3.57 ~ 6.29)	<0.001		Now	1.95 (1.17 ~ 3.24)	0.010	
Living alone	1.76 (1.14 ~ 2.71)	0.011		Drinking			**0.005**
PIR			0.605	Never	12.01 (4.24 ~ 34.01)	<0.001	
≤1.0	3.31 (1.93 ~ 5.68)	<0.001		Past	1.62 (0.63 ~ 4.14)	0.315	
1.1-4.0	3.62 (2.61 ~ 5.01)	<0.001		Now	3.29 (2.56 ~ 4.22)	<0.001	
>4.0	3.46 (2.22 ~ 5.39)	<0.001		Diabetes			0.940
BMI			0.095	Yes	3.68 (2.17 ~ 6.22)	<0.001	
<25	2.45 (1.35 ~ 4.44)	0.003		No	3.42 (2.62 ~ 4.47)	<0.001	
25-30	4.73 (3.21 ~ 6.98)	<0.001		Pre-diabetes	17.61 (0.82 ~ 375.82)	0.066	
≥30	3.52 (2.46 ~ 5.02)	<0.001		Sedentary activity			0.101
CVD			0.404	No	3.18 (2.21 ~ 4.55)	<0.001	
No	3.44 (2.68 ~ 4.42)	<0.001		Yes	3.87 (2.85 ~ 5.27)	<0.001	
Yes	4.51 (2.24 ~ 9.10)	<0.001					

Bold values indicate statistically significant differences (P-value < 0.05).

## 4 Discussion

Our investigation primarily investigated the relevance between the AIP and the prevalence of TD in adult males using the NHANES database. The research involved in a sample size of 4,115 individuals. Weighted multifactorial logistic regression analysis revealed, after all covariates were controlled, a positive and statistically significant relationship between the prevalence of TD and the AIP in adult males. Furthermore, the RCS curves revealed a non-linear positive correlation between TD and the AIP in adult males. Additionally, the prevalence of TD progressively increased with the increasing AIP quartiles in adult males. The subgroup analyses revealed that the relationship between AIP and TD might be altered because of marital status, CKD, smoking, and alcohol consumption. Future research should also explore the complex interplay between the AIP, testosterone, and comorbid conditions such as chronic kidney disease and diabetes mellitus, where dyslipidemia and endocrine dysfunction may create synergistic detrimental effects on male health outcomes.

The positive correlation of the AIP with the incidence of TD varied among certain subgroups. Different marital statuses may lead to different psychological and physiological stresses that may affect the stability of testosterone levels. It has been revealed that men living with a partner have lower testosterone levels compared to unmarried men (31), a result that leads to an unstably positive synergistic relationship between the atherosclerosis index and testosterone deficiency across marital status. Meanwhile, a broad spectrum of studies has underscored that smoking has an effect on sperm density, viability, vigor, and morphology; seminal plasma zinc concentration; and sperm DNA damage in men, with the sperm DNA fracture index being higher in smoking than in non-smoking groups and sperm DNA fragmentation being increased. This suggests that smoking may affect testosterone synthesis and sperm quality by increasing DNA damage (32). Alcohol consumption may lead to abnormal liver function and affect the metabolism of sex hormone-binding globulin, which in turn affects the bioavailability of testosterone (33). In summary, smoking and alcohol consumption may combine to destabilize the association between AIP and testosterone deficiency by affecting the endocrine system, increasing oxidative stress, and affecting liver function. In addition, changes in renal function directly affect hormone metabolism and excretion, which may influence testosterone levels and lead to unstable outcomes. These findings raise important clinical questions about whether targeted interventions such as dietary modifications, lifestyle changes, or pharmacologic lipid-lowering therapies could serve as adjunct strategies for managing androgen deficiency in metabolic syndrome populations, a hypothesis requiring verification through randomized controlled trials. Similarly, lifestyle interventions that reduce visceral adiposity and insulin resistance could ameliorate both the AIP and testosterone deficiency through shared metabolic pathways. Randomized controlled trials are urgently needed to evaluate whether AIP-lowering strategies serve as adjunct therapies for managing androgen deficiency in high-risk populations, particularly those with metabolic syndrome or CKD.

An array of investigations have revealed that TD is a common condition. It has a significant influence on the quality of life and healthy condition of the patient, as well as a remarkable influence on the psychological wellbeing of individuals. Nevertheless, the underlying pathological processes of TD remain incompletely understood. Although the occurrence of TD in the adults has remained at the same level in recent years, the overall incidence and public health impact of the condition remains quite significant given the rising population size, which may indirectly reflect trends in the development of TD in other countries around the globe. This exacerbates the strain on healthcare systems and increases explicit and implicit medical costs connected to the condition. In addition, TD is connected to a variety of chronic diseases, including cardiovascular and disease kidney disease, further increasing the need for public health interventions. An elevated AIP is a key indicator of lipid metabolite interactions and is associated with the incidence of TD, a relationship that may involve multiple mechanisms. We introduced the AIP, which combines triglyceride to HDL-C ratios, and concluded that it more accurately reflects an individual’s metabolic health status.

Several mechanisms may explain the positive relationship between the incidence of TD and AIP in adults with diabetes. These include several aspects such as dyslipidemia, insulin resistance, inflammatory response, and hepatic lipid metabolism (34–38). Initially, research demonstrated that testosterone significantly contributes to the regulation of lipid metabolism in male individuals. Serum testosterone levels were positively correlated with HDL-C and inversely related to low-density lipoprotein cholesterol (LDL-C), triglycerides, and total cholesterol (39, 40). Correlation studies indicate that higher levels of testosterone in the blood are associated with increased levels of HDL-C, while they are inversely related to the concentrations of low-density lipoprotein cholesterol (LDL-C), triglycerides, and total cholesterol.

This implies that endogenous testosterone may be a favorable factor in maintaining the balance of lipid metabolism in men. When testosterone levels are low, this balance is disrupted, resulting in increased levels of TG and non-HDL-C and decreased levels of HDL-C. This implies that testosterone deficiency may contribute to an elevated AIP by affecting lipid levels, specifically by increasing harmful lipid components (TG and non-HDL-C) and decreasing beneficial lipid components (HDL-C) (41). At the same time, testosterone deficiency results in a compromised increase of HDL-C triggered by its conversion to estradiol by aromatase, which also leads to an increased AIP (42). Furthermore, evidence has demonstrated that retinol-binding protein 4 (RBP4) is crucial for managing insulin resistance. Notably, in a murine model exhibiting diminished testosterone activity, there was a marked increase in RBP4 protein concentrations, potentially linking it to the onset of insulin resistance (43–45). Furthermore, insulin resistance could contribute to the activation of the fatty acid synthesis pathway in adipocytes, which promotes fatty acid synthesis and fat accumulation while inhibiting fatty acid oxidation, leading to dyslipidemia (46), which in turn affects AIP levels. In addition, research has demonstrated a strong correlation between testosterone deficiency and increased levels of inflammatory indicators, including high-sensitivity C-reactive protein, interleukin-6, and tumor necrosis factor-alpha (47). This implies that TD may be associated with increased levels of inflammation in the body, while the AIP is dependent on the logarithm of the ratio of plasma TG to HDL-C, which reflects the size of oxidized LDL particles in serum. Inflammatory factors can affect lipid metabolism (48), particularly by increasing the levels of oxidized low-density lipoprotein (oxLDL), which may lead to elevated AIP levels. Ultimately, testosterone may alter the genetic expression linked to the formation and release of lipoproteins, including the scavenger receptor B1 (SR-B1). Hepatic lipase expression is upregulated in hepatocytes. This enzyme catalyzes the breakdown of phospholipids on HDL-C particles and facilitates the selective uptake of HDL-C via SR-B1, which promotes retrograde cholesterol transport and exerts an antiatherosclerotic effect (49), thus leading to the correlation between an elevated AIP and testosterone deficiency. The biological mechanisms underlying this association warrant further investigation, particularly regarding how altered lipid metabolism may impact cell function, steroidogenic enzyme activity, or androgen receptor signaling through inflammatory or oxidative stress pathways.

According to studies in recent years, the current rate of TD in the global population continues to rise, affecting people’s physical and mental health in various ways. Lipid metabolism is closely associated with TD problems. Our investigation confirms a novel demonstration of a significant relationship between the AIP and the occurrence of TD in adult males, which has significant implications for the diagnosis and treatment of TD. This research exhibits several strengths. Based on the NHANES database, the investigation is primarily a cross-sectional investigation to research the correlation between the AIP and TD in adult males. At the same time, our study is representative because it contains basic data from a large number of respondents in the US. However, it is worth noting that we still have some limitations. First, some of the information in the NHANES database was obtained through self-reporting by the respondents, including disease information. Self-reporting bias could lead to imprecise answers, which may affect the accuracy of the results. Second, this investigation could not establish a direct sequential association between TD and the AIP in adult males, but only inferred a correlation, due to it being a cross-sectional study. Longitudinal studies tracking lipid profiles and hormonal changes over time are needed to establish temporal relationships and clarify potential causal pathways. The diagnosis of testosterone deficiency relied solely on a single serum testosterone measurement without clinical symptom assessment. Diurnal variation in testosterone levels and the absence of symptom data may have introduced misclassification bias. Future studies should incorporate repeated measures, standardized morning blood draws, and validated symptom questionnaires. In conclusion, this study suggests there is a positive significant relationship between the AIP and the risk of TD in adult males.

## 5 Conclusion

The research revealed a positive significant relationship between the AIP and TD in American adult males. In adult males, a higher AIP was correlated with a higher occurrence of TD. This finding suggests that lipid metabolism may be an influencing factor for TD. The clinical importance of our research is that assessing the AIP may help identify those at higher risk for TD. Incorporation of the AIP into common clinical assessment could contribute to early detection of TD and direct personalized care strategies.

## Data Availability

The original contributions presented in the study are included in the article/supplementary material. Further inquiries can be directed to the corresponding author.
